# Vaccination with 10-valent pneumococcal conjugate vaccine in infants according to HIV status

**DOI:** 10.1097/MD.0000000000005881

**Published:** 2017-01-13

**Authors:** Shabir A. Madhi, Anthonet Koen, Lisa Jose, Nadia van Niekerk, Peter V. Adrian, Clare Cutland, Nancy François, Javier Ruiz-Guiñazú, Juan-Pablo Yarzabal, Marta Moreira, Dorota Borys, Lode Schuerman

**Affiliations:** a Medical Research Council: Respiratory and Meningeal Pathogens Research Unit; b Department of Science and Technology/National Research Foundation: Vaccine Preventable Diseases, University of the Witwatersrand, Johannesburg, South Africa; c National Institute for Communicable Diseases: a Division of the National Health Laboratory Service, Johannesburg, South Africa; d GSK, Wavre, Belgium.

**Keywords:** 10-valent pneumococcal conjugate vaccine, acquired immunodeficiency syndrome, Africa, HIV, immunization, infant, safety

## Abstract

**Background:**

Phase III, open-label, single-center, controlled study in South Africa (ClinicalTrials.gov: NCT00829010) to evaluate immunogenicity, reactogenicity, and safety of the 10-valent pneumococcal non-typeable *Haemophilus influenzae* protein D conjugate vaccine (PHiD-CV) in human immunodeficiency virus (HIV)-infected (HIV+), HIV-exposed-uninfected (HEU), and HIV-unexposed-uninfected (HUU) children.

**Methods:**

Children stratified by HIV status received PHiD-CV primary vaccination (age 6/10/14 weeks; coadministered with routine childhood vaccines) and booster dose (age 9–10 months). Immune responses, assessed using enzyme-linked immunosorbent and functional assays, and safety were evaluated up to 14 months post-booster.

**Results:**

Of 83, 101, and 100 children enrolled in HIV+, HEU, and HUU groups, 70, 91, and 93 were included in according-to-protocol immunogenicity cohort. For each vaccine-serotype, percentages of children with antibody concentrations ≥0.2 μg/mL were ≥97% 1 month post-primary vaccination and ≥98.5% 1 month post-booster (except for 6B and 23F at both timepoints). Post-primary vaccination, functional antibody responses were lower in HIV+ children: for each vaccine-serotype, percentages of children with opsonophagocytic activity (OPA) titres ≥8 were ≥72%, ≥81%, and ≥79% for HIV+, HEU, and HUU children. Post-booster, ≥87% of children in each group had OPA titres ≥8. Reactogenicity was similar across groups. Thirty one (37%) HIV+, 25 (25%) HEU, and 20 (20%) HUU children reported ≥1 serious adverse event. Five HIV+ and 4 HEU children died. One death (sudden infant death syndrome; HEU group; 3 days post-dose 1) was considered potentially vaccine-related.

**Conclusion:**

PHiD-CV was immunogenic and well-tolerated in HIV+, HEU, and HUU children, and has the potential to provide substantial benefit irrespective of HIV infection status.

## Abstract 2

Supplemental Digital Content is available in the text

## 1 Introduction

*Streptococcus pneumoniae* is a leading cause of bacterial invasive disease, upper respiratory tract infections and pneumonia in young children, with a higher risk in human immunodeficiency virus (HIV)-infected and HIV-exposed-uninfected (HEU) children.^[1,2]^ Despite significant declines in respiratory disease and invasive pneumococcal disease (IPD) morbidity after introduction of highly active antiretroviral therapy (HAART) in Africa, HIV-infected (HIV+) children remain a high-risk group for respiratory illnesses and IPD.^[3]^

After global implementation of Prevention of Mother-to-Child Transmission programmes, most children born to HIV-infected women will not be infected themselves. Nevertheless, these HEU children are at higher risk of morbidity and mortality than children born to HIV-uninfected mothers.^[4–6]^ Moreover, HEU children responded differently to Bacille Calmette-Guérin vaccine than HIV-unexposed-uninfected (HUU) children, indicating potential alterations in their immune systems.^[7]^

Pneumococcal conjugate vaccines (PCVs) using the non-toxic cross-reactive mutant of diphtheria toxin CRM_197_ as carrier protein have been shown to prevent a substantial burden of pneumococcal disease in HIV-infected children not managed with HAART, despite lower vaccine efficacy and immune response compared to HIV-uninfected infants.^[8–10]^ An ecological study from South Africa reported 85% reduction in the incidence of IPD caused by 7-valent PCV (PCV7) serotypes among HIV-uninfected children below 2 years of age, within 4 years after PCV introduction. Among HIV-infected children younger than 2 years, where the rate of disease was 25 times higher than among HIV-uninfected children, PCV7-serotype IPD declined by 86%. A reduction of approximately 55% of PCV7-serotype IPD was attributable to vaccination.^[11]^

For the 10-valent pneumococcal non-typeable *Haemophilus influenzae* protein D conjugate vaccine (PHiD-CV) which uses non-typeable *H. influenzae* protein D as carrier protein for 8 of its 10 pneumococcal serotypes, we present here results from the first study assessing its immunogenicity and safety in HIV-infected and HIV-exposed infants.

## 2 Patients and methods

### 2.1 Study design and participants

This phase III, open, single-center, controlled study was conducted in South Africa between February 2009 and June 2012. The study was partially randomized; only HUU children were randomized to assess different PHiD-CV vaccination schedules (these results will be reported elsewhere).

Eligible participants were infants between and including 6 to 10 weeks of age at first vaccination, without any known or suspected health problems (other than HIV infection or exposure) that would contraindicate initiation of routine immunizations. Infants were excluded if they had moderately or severely symptomatic HIV infection (stages 3 and 4 according to the World Health Organization [WHO] classification^[12]^). The study included 3 populations of children, based on HIV status of mother and child: HIV+ (HIV-positive mother, and infant confirmed as HIV-infected by HIV deoxyribonucleic acid polymerase chain reaction [DNA-PCR] at screening and HIV viral load test at visit 1), HEU (HIV-positive mother, and infant confirmed as HIV-uninfected by HIV DNA-PCR at screening), and HUU (HIV-negative mother and infant, confirmed as HIV-uninfected by HIV enzyme-linked immunosorbent assay [ELISA] after 24 weeks of gestation for the mother and at visit 1 for the infant). Detailed inclusion/exclusion criteria and HIV assessments can be found in Supplemental Digital Content 1. The study comprised 10 visits (Fig. 1); for each participant, study duration was approximately 24 months. For HIV+ children, CD4 percentages, viral load, WHO HIV clinical staging, and antiretroviral therapy (ART) were tabulated when data were available.

**Figure:**
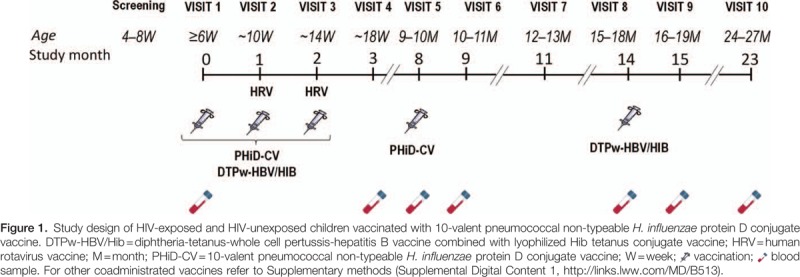


The study was conducted according to the principles of Good Clinical Practice and the Declaration of Helsinki, and with the approval of an independent ethics committee (Wits Human Research Ethics Committee). Written informed consent was obtained from the parent(s) or legally acceptable representative(s) of each child before any study-specific procedures. An independent data monitoring committee provided oversight of the study by assessing potential treatment harm and reviewing all-cause mortality and morbidity.

This study has been registered at www.clinicaltrials.gov (NCT00829010). A protocol summary is available at www.gsk-clinicalstudyregister.com (study ID: 111634).

### 2.2 Study objectives

The primary objective was to evaluate immune responses to PHiD-CV at 1 month after 3-dose primary vaccination in HIV+, HEU, and HUU infants. Secondary objectives included assessment of antibody persistence approximately 6 months post-primary vaccination, immune responses to a PHiD-CV booster dose, antibody persistence 14 months post-booster, and evaluation of safety and reactogenicity. Other secondary objectives, including assessment of nasopharyngeal carriage and immunogenicity and safety of coadministrated vaccines, will be reported elsewhere.

### 2.3 Treatment allocation and study vaccines

Treatment allocation at investigator site was performed using a central randomization and treatment allocation system on the internet (SBIR); randomization was only performed for HUU children, to receive different PHiD-CV vaccination schedules.

Infants were stratified into 3 groups according to their HIV status (HIV+, HEU, HUU). HIV+ and HEU children received 3 primary doses of PHiD-CV (*Synflorix*^TM^; GSK Vaccines, Belgium) at 6, 10, and 14 weeks of age, and a booster dose at 9 to 10 months of age. PHiD-CV consists of 1 μg capsular polysaccharide for serotypes 1, 5, 6B, 7F, 9V, 14, and 23F, and 3 μg for serotype 4, each individually conjugated to non-typeable *H. influenzae* protein D, and 3 μg of capsular polysaccharide of serotypes 18C and 19F conjugated to tetanus and diphtheria toxoids, respectively.

HUU children were randomized (1:1:1) into 3 subgroups with different PHiD-CV vaccination schedules: 3 primary doses plus booster (3+1), 3 primary doses without booster (3+0), and 2 primary doses plus booster (2+1). A randomization blocking scheme was used to ensure that balance between treatments was maintained. While results for the groups receiving 3 primary PHiD-CV doses are presented here, results on different dosing schedules in HUU groups will be reported elsewhere. Post-primary immunogenicity results of pooled HUU (3+1) and HUU (3+0) groups, referred to as HUU (3+1/3+0), were used for exploratory between-groups assessment as children in these groups had the same HIV status and primary vaccination regimen. Other routinely coadministered vaccines, including diphtheria-tetanus-whole cell pertussis-hepatitis B vaccine combined with lyophilized Hib tetanus conjugate vaccine (DTPw-HBV/Hib), human rotavirus vaccine, measles and oral polio vaccine (OPV), are detailed in Supplemental Digital Content 1.

### 2.4 Immunogenicity assessment

Blood samples for serology assessment were taken pre-vaccination, 1 month post-primary vaccination, pre-booster, and 1 and 14 months post-booster of PHiD-CV, as well as pre- and 1 month post-booster vaccination of coadministered vaccines.

Immunoglobulin G (IgG) antibodies against vaccine-serotypes and vaccine-related serotypes 6A and 19A (belonging to the same serogroup as vaccine-serotypes) were quantified using GSK's 22F-ELISA (cut-off: 0.05 μg/mL). Immune response was described in terms of percentages of children with IgG concentrations ≥0.2 μg/mL (equivalent to antibody concentrations ≥0.35 μg/mL measured by the non-22F ELISA of the WHO reference laboratory^[13]^).

Opsonophagocytic activity (OPA) was measured by a pneumococcal killing assay using an HL-60 cell line (cut-off titre: 8).^[14]^ Results are presented as the reciprocal of the dilution of serum (opsonic titre) able to sustain 50% killing of pneumococci under assay conditions.

Anti-protein D antibodies were quantified using an ELISA assay developed by GSK (cut-off: 100 ELISA Units/mL).

### 2.5 Safety assessment

Solicited local and general adverse events (AEs), which are detailed in Supplemental Digital Content 1, were recorded within 4 days post-vaccination, and unsolicited AEs within 31 days post-vaccination. Serious adverse events (SAEs), defined as any medical occurrence that resulted in death, disability, or incapacity, was life-threatening, required hospitalization or was considered serious by the investigator, were recorded during the entire study.

### 2.6 Statistical analysis

Per group, 100 children were planned to be enrolled, resulting in 80 evaluable children in the according-to-protocol cohort with an exclusion rate of 20%. The study had no confirmatory objectives which would drive its sample size. All objectives including the primary objective were descriptive.

Safety analyses were performed on the total vaccinated cohort, including all children with ≥1 administered vaccine dose. Immunogenicity analyses were performed on the according-to-protocol immunogenicity cohort, comprising vaccinated children who met all eligibility criteria, complied with the protocol-defined procedures, and with assay results available for antibodies against ≥1 study vaccine antigen.

Geometric mean antibody concentrations (GMCs) and geometric mean OPA titres (GMTs) were calculated for each serotype or antigen with 95% confidence intervals (CIs) by taking the anti-log of the mean of the log antibody concentration or titre transformations. Antibody concentrations and OPA titres below assay cut-offs were given an arbitrary value of half the cut-off for GMC and GMT calculations. Percentages of children with serological results above respective thresholds were calculated with 95% CIs.

In exploratory group comparisons, potential group differences were highlighted when the 95% CI on GMC and GMT ratios excluded 1, or the 95% CI on differences in percentage of children with antibody concentrations or OPA titres above cut-offs excluded 0. GMC and GMT ratios with 95% CIs were obtained using an analysis of variance model (including only the vaccine group as fixed effect) on logarithm-transformed antibody concentrations and OPA titres. No adjustment for multiplicity of comparisons or baseline serology was performed.

Statistical analyses were performed using Statistical Analysis System (SAS) Drug and Development web portal version 3.5, SAS version 9.2, and Proc StatXact-8.1 procedure on SAS (SAS Institute Inc., Cary, NC, USA).

## 3 Results

### 3.1 Study participants

**Figure 2:**
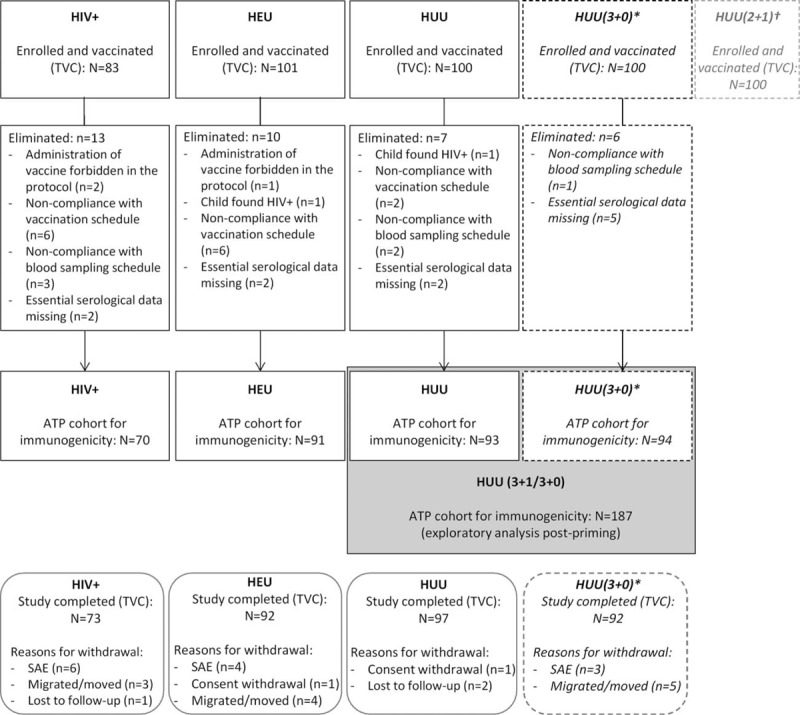
Participant flow of HIV-exposed and HIV-unexposed children vaccinated with 10-valent pneumococcal non-typeable *H. influenzae* protein D conjugate vaccine. ATP = according-to-protocol; N  = number of children; n = number with the characteristic; HEU = HIV-exposed-uninfected; HIV+ = HIV-infected; HUU = HIV-unexposed-uninfected; SAE = serious adverse event; TVC = total vaccinated cohort. ∗Only primary and first persistence analysis presented here. †Results presented elsewhere. Thirteen children with subject numbers were not vaccinated; they did not contribute to results.

All groups reached the expected number of 100 participants, except for the HIV+ group (83 participants) (Fig. 2). All groups were comparable in terms of age, ethnicity, and sex. Mean body weight throughout the study tended to be lower in the HIV+ group (see Supplemental Digital Content 2). The majority of HIV+ children maintained a good CD4 cell count during the study (see Supplemental Digital Content 3). HAART were reported by 72% to 96% of HIV+ children during the primary series and by 100% post-booster, leading to a decrease in viral load over time (see Supplemental Digital Content 3). Four infants initially considered HIV+ were reallocated as HEU, because they were born to an HIV-positive mother, confirmed as being HIV-infected by HIV DNA-PCR at screening, but had undetectable viral load at visit 1 (3 children) or undetermined HIV status at retesting (1 child). One child, who was wrongly randomized in the HUU (3+1) group, was also considered HEU for the analysis.

### 3.2 Immunogenicity results

#### 3.2.1 Post-primary vaccination results

One month post-dose 3, for each vaccine-serotype, ≥97% of children had antibody concentrations ≥0.2 μg/mL, except for 6B (≥80%) and 23F (≥89%). For vaccine-related serotypes, percentages ranged from 28% to 33% for 6A, and from 38% to 57% for 19A. All children, except 1 in the HEU group, had measurable antibody concentrations against protein D (Table 1).

**Table 1:**
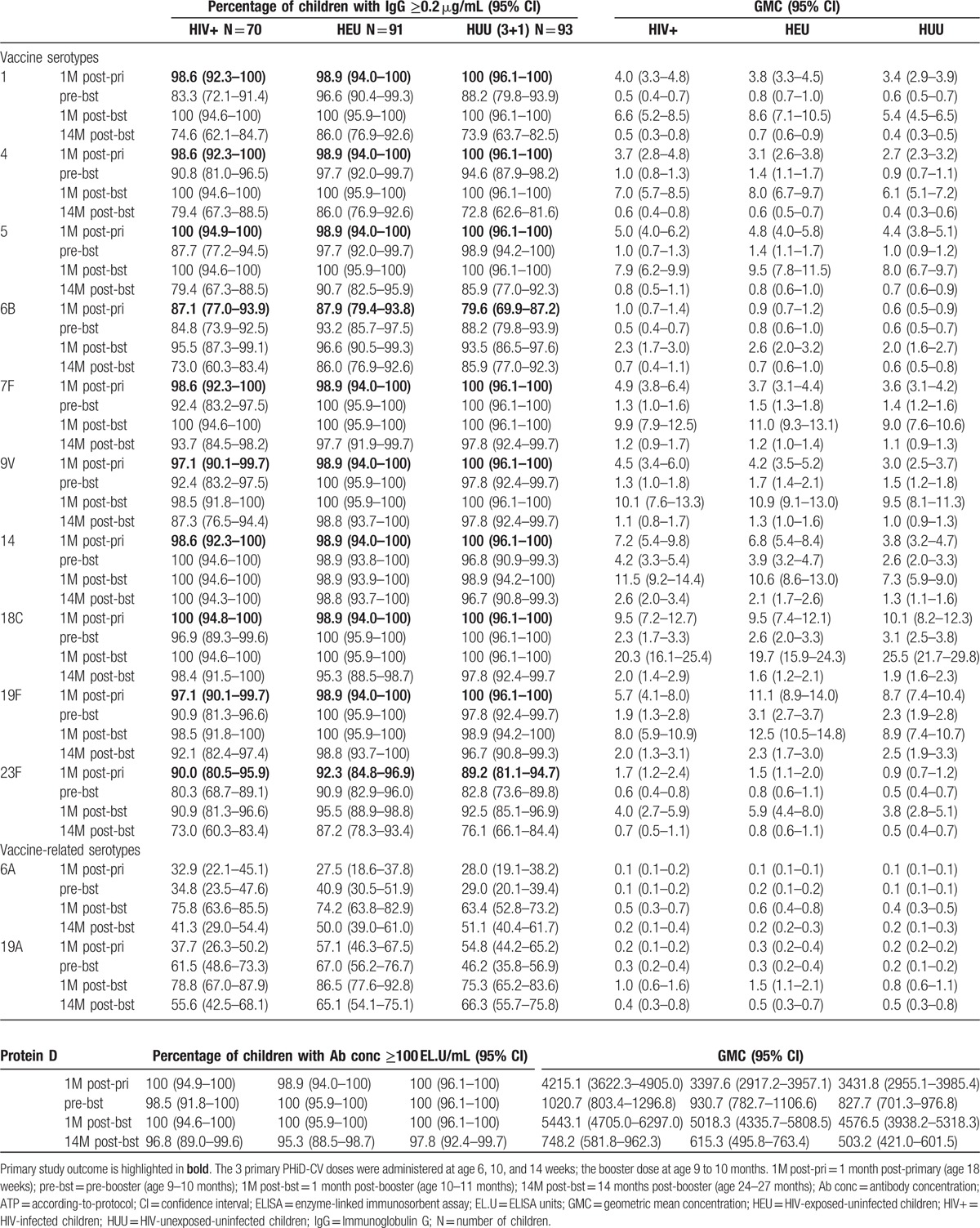
Serotype-specific pneumococcal and protein D antibody responses (ATP cohort for immunogenicity).

**Figure 3:**
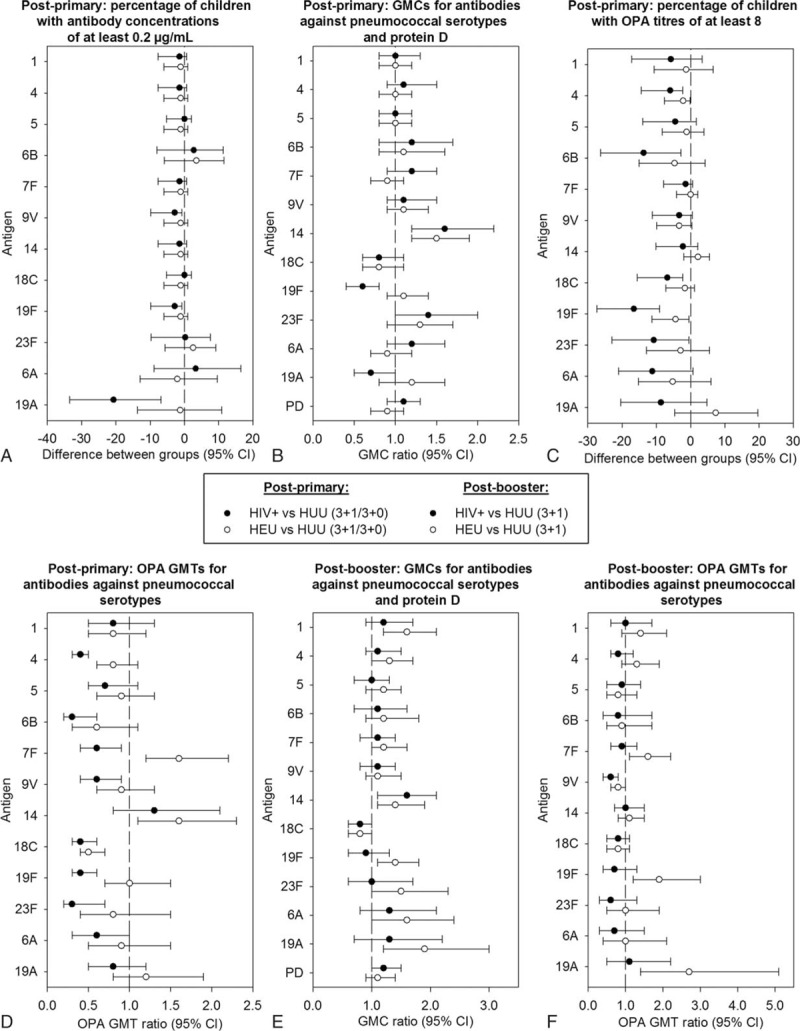
Exploratory between-group comparisons of immunogenicity post-primary and post-booster vaccination (ATP immunogenicity cohort). Differences: HIV+ or HEU minus HUU; ratios: HIV+ or HEU divided by HUU. Post-primary, the HUU group consists of the pooled HUU (3+1/3+0) groups (panels A–D), and post-booster, the HUU group consists of the HUU (3+1) group (panels E–F). ATP = according-to-protocol; CI = confidence interval; GMC = geometric mean concentration; GMT = geometric mean titer; HEU = HIV-exposed-uninfected; HIV+ = HIV-infected; HUU = HIV-unexposed-uninfected; OPA = opsonophagocytic activity; PD = protein D.

Exploratory group comparisons did not suggest any differences between HIV+ and HUU (3+1/3+0) groups, except for percentages of children with antibody concentrations ≥0.2 μg/mL for serotypes 9V, 19F, and 19A (Fig. 3A) and antibody GMCs for serotype 19F, which tended to be lower in the HIV+ group, and antibody GMCs for serotype 14, which tended to be higher in the HIV+ group (Fig. 3B). Differences between HEU and HUU (3+1/3+0) groups were not observed, except for antibody GMCs for serotype 14, which appeared higher in the HEU group (Fig. 3B).

For each vaccine-serotype, ≥82% of children had OPA titres ≥8, except for 6B (≥72%) and 23F (77% for HIV+) (Table 2). Percentages of children with OPA titres ≥8 tended to be lower in the HIV+ group; exploratory comparisons suggested differences between HIV+ and HUU (3+1/3+0) groups for serotypes 4, 6B, 18C, 19F, and 23F (Fig. 3C). Moreover, OPA GMTs seemed lower in the HIV+ than HUU (3+1/3+0) group for all serotypes except 1, 5, 14, and 19A (Fig. 3D). Differences between HEU and HUU (3+1/3+0) groups were not observed for most serotypes, except for percentages of children with OPA titres ≥8 for serotypes 4 and 19F (Fig. 3C) and OPA GMTs for serotype 18C, which tended to be lower in the HEU group, and OPA GMTs for serotypes 7F and 14, which tended to be higher in the HEU group (Fig. 3D).

**Table 2:**
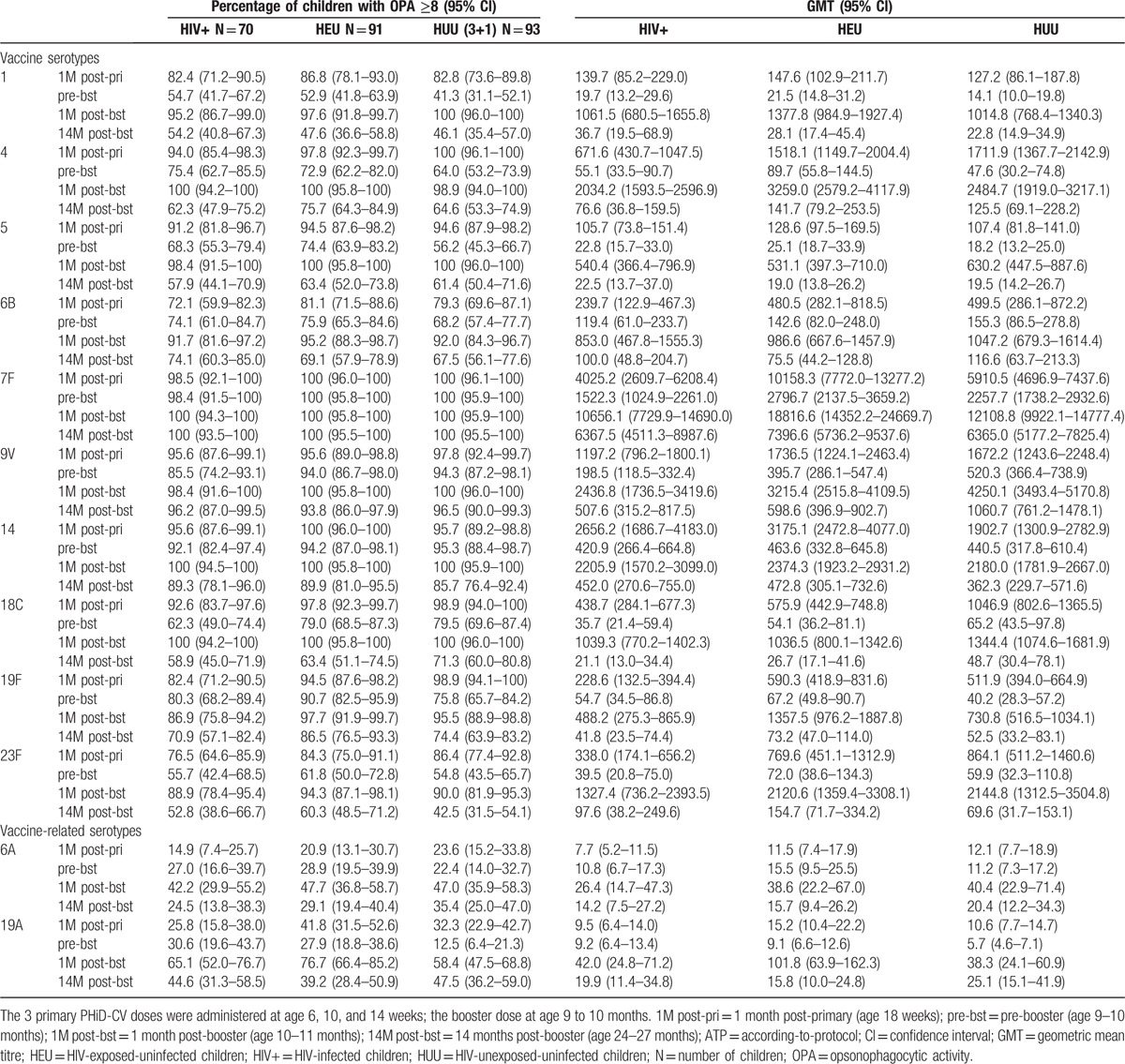
Functional immune response assessed by opsonophagocytic activity (OPA) assay (ATP cohort for immunogenicity).

At 6 months post-primary vaccination, for each vaccine-serotype, ≥80%, ≥91%, and ≥83% of children had antibody concentrations ≥0.2 μg/mL, and ≥55%, ≥53%, and ≥41% had OPA titres ≥8 in HIV+, HEU, and HUU (3+1) group, respectively (Tables 1 and 2). All children except 1 in the HIV+ group had measurable antibodies against protein D (Table 1).

Post-primary and pre-booster vaccination antibody concentrations and OPA titres for the HUU (3+1/3+0) group are presented in Supplemental Digital Content 4.

#### 3.2.2 Post-booster vaccination results

For each vaccine-serotype, ≥99% of children had antibody concentrations ≥0.2 μg/mL 1 month post-booster, except for 6B (≥94%) and 23F (≥91%). This percentage ranged from 63%–76% and 75%–87% for vaccine-related serotypes 6A and 19A, respectively. All children had measurable antibodies against protein D (Table 1).

Exploratory comparisons suggested no differences between HIV+ and HUU (3+1) groups, except for antibody GMCs for serotype 14 which tended to be higher in HIV+ children. No differences were observed between HEU and HUU (3+1) groups, except for antibody GMCs for serotypes 1, 14, 19F, and 19A which tended to be higher in the HEU group (Fig. 3E).

For each vaccine-serotype, ≥87% of children had OPA titres ≥8 (Table 2). No differences between HIV+ and HUU (3+1) groups were suggested by exploratory comparisons, except OPA GMTs for serotype 9V which appeared to be lower in the HIV+ group. OPA GMTs for serotypes 7F, 19F, and 19A appeared higher in the HEU than HUU (3+1) group (Fig. 3F).

At 14 months post-booster, for each vaccine-serotype, ≥75%, ≥86%, and ≥73% of children had antibody concentrations ≥0.2 μg/mL, and ≥53%, ≥48%, and ≥43% had OPA titres ≥8 in the HIV+, HEU, and HUU (3+1) group, respectively (Tables 1 and 2). In each group, ≥95% of children had measurable antibodies against protein D (Table 1).

### 3.3 Safety results

In all groups, pain was the most frequently reported solicited local symptom at the PHiD-CV injection site. Post-primary vaccination, the most common solicited general symptom in all groups was irritability (see Supplemental Digital Content 5). During the 4-day post-vaccination period, antipyretic treatments were taken after 31%, 33%, and 48% of all primary doses, and after 23%, 27%, and 29% of booster doses in the HIV+, HEU, and HUU (3+1) group, respectively.

Incidences of unsolicited symptoms were in similar ranges in the HIV+, HEU, and HUU (3+1) groups (88%, 91%, and 93% after all primary doses; 46%, 49%, and 51% post-booster), with cough being the most common in all groups.

At least 1 SAE was reported by 31 (37%) HIV+, 25 (25%) HEU, and 20 (20%) HUU (3+1) children. Nine children died: 5 in the HIV+ group (bronchopneumonia; convulsion; unknown cause; sudden death possibly due to pneumonia; and sudden death due to unknown cause) and 4 in the HEU group (sudden infant death syndrome [SIDS]; measles and bronchopneumonia; diarrhoea; and encephalopathy, gastroenteritis, kwashiorkor, anaemia, and renal impairment). One of these fatalities (SIDS in the HEU group) was considered to be possibly related to vaccinations due to its temporal association (3 days post-dose 1 of PHiD-CV coadministered with DTPw-HBV/Hib and OPV). Two other non-fatal SAEs were also considered by the investigator to be temporally related to vaccinations (gastroenteritis in an HIV+ child and febrile convulsion in an HUU child; both resolved).

## 4 Discussion

This is the first report on PHiD-CV in HIV-infected and HIV-exposed-uninfected children. Exploratory analyses did not suggest differences in immune response between HIV+ and HUU children for most vaccine-serotypes post-primary vaccination and for all vaccine-serotypes post-booster, or between HEU and HUU children both post-primary and post-booster vaccination, even if post-booster antibody GMCs for serotypes 1, 14, 19F, and 19A tended to be higher in the HEU group.

Exploratory analyses indicated lower OPA GMTs in HIV+ compared with HUU children for most vaccine-serotypes post-primary vaccination. However, these differences should be interpreted cautiously because no adjustment was made for multiplicity of comparisons or baseline serology, the study was not powered for group comparisons to draw conclusions, and the clinical relevance of such differences in OPA titres is unknown. Post-booster OPA GMTs were in similar ranges in HIV+ and HUU children for all vaccine-serotypes, except for 9V.

Although HEU infants may have lower maternal antibody acquisition, similar or even better immune responses have been reported in these children post-primary vaccination;^[15–17]^ higher antibody levels in HEU children could be due to less interference by maternal antibodies. However, concerns have risen about the waning of immunity in HEU children, as shown for measles vaccination.^[17]^ Here, antibody GMCs in HEU and HUU children were in similar ranges for all vaccine-serotypes at 14 months post-booster.

Immune responses in HIV+ children with percentages of CD4+ cells ≥25% (signifying immuno-competence) have been previously assessed for PCV7;^[16,18]^ higher antibody concentrations were observed post-primary vaccination in HIV+ children on ART than in HUU children, with similar OPA titres (except for 19F and 23F).^[16]^

The strengths of our study are that we did not pre-select for CD4+ level, although moderately and severely clinically symptomatic children were excluded, and HAART were used as part of standard-of-care and according to the latest WHO recommendations, making our results more generalizable. A past study on 9-valent PCV showed waning of immunity and protection at 5 to 6 years of age in HIV+ children who were not on ART.^[9]^ Here, antibody GMCs for all serotypes were in similar ranges in HIV+ and HUU children at 14 months post-booster; antibody persistence follow-up will continue up to 5 years. Immune response in HIV+ children and adolescents has also been assessed for 13-valent PCV; children with perinatally-acquired HIV had mounted robust serotype-specific IgG responses 1 month after a single 13-valent PCV dose, persisting up to 6 months for most serotypes.^[19]^

Reactogenicity in our study was similar to that previously observed in African settings with DTPw-HBV/Hib co-administration.^[20–22]^ Separate assessment of PHiD-CV safety was limited to local reactogenicity only; systemic AEs were also recorded albeit after coadministration of other vaccines. The obtained results are relevant as coadministration of various vaccines is common in vaccination programmes. Out of the 284 children, 9 died, which was not unexpected considering the underlying conditions of these children and the reported mortality rates in South Africa.^[23,24]^

One case of SIDS in an HEU infant occurred 3 days after the first doses of PHiD-CV coadministered with DTPw-HBV/Hib and OPV; no autopsy was performed, and the mother reported that the infant had been well post-vaccination. Due to its temporal association, the investigator considered that a causal association with the study vaccines or the concomitant OPV could not be excluded.

PHiD-CV vaccine efficacy against pneumococcal disease has been shown in efficacy trials,^[25–27]^ as well as in post-marketing studies,^[28–31]^ but was not evaluated here. We assessed immunogenicity in terms of percentages of children reaching antibody concentrations of 0.2 μg/mL for our 22F-ELISA (corresponding to the WHO-recommended threshold of 0.35 μg/mL); recent data suggest that this threshold may underestimate efficacy for some serotypes (6A, 6B, 18C, and 23F), while over-estimating for others (1, 3, 7F, 19A, and 19F).^[32]^ Given the comparability of immune responses between the different groups, it seems reasonable to conclude that PHiD-CV may provide protection against pneumococcal disease in HIV+ children.

Other study limitations include the absence of a confirmatory objective despite the relatively large HIV+ infant group, the long recruitment period for the HIV+ group due to successful programmes aimed at reducing mother-to-child HIV transmission, and the open design that could cause a biased reactogenicity assessment.

## 5 Conclusion

PHiD-CV was immunogenic and had a clinically acceptable safety profile in HIV+, HEU, and HUU South African infants. The PHiD-CV safety and reactogenicity profile was similar in all groups. Our results suggest that in HIV+ infants on ART, who remain at high risk for pneumococcal disease,^[3]^ PHiD-CV may confer levels of protection in similar ranges as in uninfected and unexposed infants.

Synflorix, Tritanrix-HepB/Hib, and Rotarix are trademarks of the GSK group of companies.

## Acknowledgments

The authors thank the clinical and serological laboratory teams of the GSK group of companies for their contribution to this study, in particular Sudheer Ravula (GSK) for statistical analysis, Mireille Venken (GSK), Janice Beck, Kristel Vercauteren (XPE Pharma & Science c/o GSK), and Ann Dhoest (freelance c/o GSK) for protocol and clinical report writing; Raquel Merino, Charlotte de Buck van Overstraeten, Catena Lauria (GSK), and Katleen Van Hoefs (Keyrus Biopharma c/o GSK) for global study management. The authors also thank Joke Vandewalle (XPE Pharma & Science) for drafting the manuscript; and Bart van Heertum (XPE Pharma & Science c/o GSK) for manuscript coordination.

## Supplementary Material

Supplemental Digital Content
