# Supplementary material for: Vaccination with 10-valent pneumococcal conjugate vaccine in infants according to HIV status
Source: Medicine (Baltimore). 2017 Jan 13;96(2):e5881. doi: 10.1097/MD.0000000000005881 (PMC5266190; doi:10.1097/MD.0000000000005881)
Supplement: Supplemental Digital Content [file medi-96-e5881-s001.pdf]

Vaccination with 10-valent pneumococcal conjugate vaccine in infants according to HIV status

By Shabir A. MADHI *et al.*

Supplemental Digital Content 1. Methodology

Inclusion and exclusion criteria

Eligible participants were infants between and including 6–10 weeks of age at the time of the first vaccination, who were free of any known or suspected health problems (other than human immunodeficiency virus [HIV] infection or exposure) that would contraindicate the initiation of routine immunizations, for whom the investigator believed that their parents or guardians could and would comply with the protocol requirements, and with written informed consent obtained.

Exclusion criteria included the use of any investigational or non-registered product other than the study vaccines within 30 days before the first study dose, or any planned use during the study period; previous vaccination against or history of diphtheria, tetanus, pertussis, *Haemophilus influenzae* type b, rotavirus or *S. pneumoniae*; administration of immunoglobulins or any blood products since birth or planned administration during the study period; a family history of hereditary immunodeficiency; history of allergic disease or reactions likely to exacerbated by any component of the vaccines; weight <3rd percentile at Visit 1 (except for HIV-infected infants for which the decision of enrolment was left to the investigator's discretion); and moderately or severely symptomatic HIV (World Health Organisation stages III and IV). Additionally, study entry was delayed in case of disease at the time of enrolment, and vaccination was deferred in case of gastroenteritis within 7 days preceding planned vaccination.

HIV analyses

The HIV status of infants born from an HIV-positive mother was determined by HIV deoxyribonucleic acid polymerase chain reaction (DNA-PCR) during screening at age of 4–8 weeks. For HIV-infected children, an HIV viral load test was performed at visit 1 for confirmation of the HIV status and at visit 10, using an HIV ribonucleic acid-PCR. CD4 count was performed at visits 1, 5, 8 and 10. For HIV-exposed-uninfected children, an HIV DNA-PCR was performed 2 months after breastfeeding cessation and an HIV enzyme-linked immunosorbent assay (ELISA) at visit 10. For HIV-unexposed-uninfected children, an HIV ELISA was performed at visits 1 and 10. No HIV testing was performed on the mothers within the context of this study; identification of HIV infection relied on testing performed within the PMCTC program. Infants born from an HIV-uninfected mother but with a positive ELISA test at visit 1 had a confirmatory HIV DNA-PCR test and were excluded from the according-to-protocol statistical analyses if found positive.

Co-administered vaccines

Infants were co-administered diphtheria-tetanus-whole cell pertussis-hepatitis B vaccine combined with lyophilized Hib tetanus conjugate vaccine (DTPw-HBV/Hib; *Tritanrix-HepB/Hib™*, GSK Vaccines, Belgium) at 6, 10 and 14 weeks of age, 2 doses of live oral attenuated human rotavirus vaccine (HRV; *Rotarix*™, GSK Vaccines, Belgium) at 10 and 14 weeks, and a DTPw-HBV/Hib booster at 15–18 months. As HRV was included after protocol amendment, some children did not receive the vaccine through participation in the study. The 10-valent pneumococcal non-typeable *Haemophilus influenzae* Protein D conjugate vaccine (PHiD-CV) and DTPw-HBV/Hib were administered intramuscularly in the right and left thigh,

respectively; HRV was administered orally. Additionally, oral polio vaccine (6, 10, 14 weeks and 15–18 months) and measles vaccine (9–10 and 15–18 months) were also administered as part of the national vaccination programme but were not considered as study vaccines.

Safety assessment

Fever was defined as axillary temperature ≥37.5°C. Diarrhoea was defined as ≥3 looser than normal stools per day. Symptom intensity was graded on a scale of 1 (mild) to 3 (severe). Grade 3 solicited adverse events were redness or swelling at the injection site with a diameter >30 mm; for pain, crying when the limb was moved or the limb being spontaneously painful; for fever, axillary temperature ≥39.5°C; for drowsiness, preventing normal activity; for irritability, crying that could not be comforted or prevented normal activity; for loss of appetite, not eating at all; for vomiting, ≥3 episodes of vomiting (≥1 hour after feeding) per day and for diarrhoea, ≥6 looser than normal stools per day; (vomiting and diarrhoea were only assessed post-priming).

Vaccination with 10-valent pneumococcal conjugate vaccine in infants according to HIV status

By Shabir A. MADHI *et al.*

Supplemental Digital Content 2. Demographic characteristics (TVC)

		HIV+<br>N=83	HEU<br>N=101	HUU<br>N=100
**Age: mean (SD)**	**Dose 1 (Weeks)**	6.6 (0.9)	6.3 (0.7)	6.1 (0.4)
	**Dose 2 (Weeks)**	10.8 (1.1)	10.6 (0.9)	10.2 (0.6)
	**Dose 3 (Weeks)**	15.3 (1.5)	14.8 (1.1)	14.3 (0.7)
	**Booster (Months)**	9.0 (0.2)	9.0 (0.3)	9.0 (0.2)
**Gender: n (%) female**		49 (59%)	47 (47%)	58 (58%)
**Ethnicity: African heritage: n (%)**		83 (100%)	101 (100%)	100 (100%)
**Weight (kg): mean (SD)**	**Visit 1**	4.0 (0.7)	4.6 (0.5)	4.5 (0.6)
	**Visit 5**	8.0 (1.1)	9.0 (1.2)	8.6 (1.2)
	**Visit 10**	10.9 (1.4)	12.4 (2.0)	11.6 (1.5)

HEU, HIV-exposed-uninfected children; HIV+, HIV-infected children; HUU, HIV-unexposed-uninfected children; N, number of children; n (%), number (percentage) of children with the specified characteristic; SD, standard deviation; TVC, total vaccinated cohort.

Vaccination with 10-valent pneumococcal conjugate vaccine in infants according to HIV status

By Shabir A. MADHI *et al.*

Supplemental Digital Content 3. HIV infection characteristics for the HIV+ group (TVC)

		Visit 1	Visit 5	Visit 8	Visit 10
**WHO clinical staging[11]: n (%)**		**N=83**	**N=77**	**N=75**	**N=73**
	Stage 1	83 (100%)	66 (86%)	62 (83%)	56 (77%)
	Stage 2	0	3 (4%)	6 (8%)	8 (11%)
	Stage 3	0	8 (10%)	7 (9%)	9 (12%)
**CD4 counts**		**N=74**	**N=67**	**N=62**	**N=72**
**(cells/mm³)**	Mean (SD)	1936.0 (759.6)	2240.7 (932.5)	2026.0 (780.2)	1832.4 (774.1)
	Median	1823.0	2093.0	1961.5	1758.5
**CD4 %**	Mean (SD)	34.6 (8.8)	34.8 (8.6)	33.2 (9.3)	33.7 (8.8)
	Median	35.5	35.0	33.4	34.5
**Viral load**		**N=83**			**N=73**
**RNA copies/mL**	Mean (SD)	533790.2 (295189.8)			32898.1 (114948.7)
	Median	750000.0			400.0
**n (%)**	<400	0 (0%)			17 (23%)
	≥400-<4000	4 (5%)			41 (56%)
	≥4000-<40000	2 (2%)			6 (8%)
	≥40000-<400000	19 (23%)			6 (8%)
	≥400000	52 (63%)			2 (3%)
	Missing	6 (7%)			1 (1%)

HIV+, HIV-infected children; N, number of children with available results at the specified visit; n (%), number (percentage) of children with the specified characteristic; RNA, ribonucleic acid; SD, standard deviation; TVC, total vaccinated cohort; WHO, World Health Organisation.

Vaccination with 10-valent pneumococcal conjugate vaccine in infants according to HIV status

By Shabir A. MADHI *et al.*

Supplemental Digital Content 4. Post-primary vaccination antibody concentration (22F-ELISA) and functional immune response (OPA) for the pooled HUU (3+1/3+0) group (ATP cohort for immunogenicity)

		% children with IgG ≥0.2 μg/mL (95% CI)	GMC (95% CI)	% children with OPA ≥8 (95% CI)	GMT (95% CI)
**Vaccine serotypes**					
1	1M post-pri	100 (98.0–100)	4.0 (3.5–4.4)	88.1 (82.6–92.4)	184.5 (143.2–237.7)
	pre-bst	89.8 (84.5–93.7)	0.7 (0.6–0.8)	50.5 (43.1–58.0)	20.5 (15.7–26.6)
4	1M post-pri	100 (98.0–100)	3.2 (2.8–3.6)	100 (98.0–100)	1798.6 (1549.1–2088.3)
	pre-bst	94.6 (90.3–97.4)	1.0 (0.9–1.2)	67.2 (59.8–74.1)	52.9 (38.3–73.1)
5	1M post-pri	100 (98.0–100)	5.0 (4.5–5.6)	95.7 (91.7–98.1)	142.3 (117.9–171.8)
	pre-bst	97.8 (94.6–99.4)	1.3 (1.1–1.4)	63.9 (56.4–70.9)	24.6 (19.4–31.1)
6B	1M post-pri	84.4 (78.4–89.3)	0.8 (0.7–1.0)	85.8 (79.9–90.5)	776.7 (548.3–1100.5)
	pre-bst	90.9 (85.8–94.6)	0.7 (0.6–0.8)	75.7 (68.7–81.8)	214.5 (146.2–314.5)
7F	1M post-pri	100 (98.0–100)	4.2 (3.7–4.6)	100 (98.0–100)	6355.7 (5354.5–7544.2)
	pre-bst	100 (98.0–100)	1.5 (1.4–1.7)	100 (98.0–100)	2143.8 (1815.6–2531.3)
9V	1M post-pri	100 (98.0–100)	4.0 (3.5–4.5)	98.9 (96.1–99.9)	1923.5 (1595.4–2319.2)
	pre-bst	97.3 (93.8–99.1)	1.6 (1.4–1.9)	94.9 (90.6–97.7)	494.8 (389.5–628.7)
14	1M post-pri	100 (98.0–100)	4.5 (3.9–5.2)	97.8 (94.5–99.4)	2048.3 (1615.6–2596.8)
	pre-bst	96.8 (93.1–98.8)	2.8 (2.4–3.4)	93.1 (88.3–96.4)	362.1 (279.5–469.2)
18C	1M post-pri	100 (98.0–100)	11.5 (9.8–13.5)	99.5 (97.0–100)	1122.7 (951.1–1325.4)
	pre-bst	100 (98.0–100)	3.4 (2.9–4.0)	78.4 (71.6–84.2)	69.4 (52.4–92.0)

19F	1M post-pri	100 (98.0–100)	9.8 (8.7–11.0)	98.9 (96.1–99.9)	576.5 (473.0–702.6)
	pre-bst	98.4 (95.4–99.7)	2.4 (2.1–2.8)	77.8 (71.0–83.6)	46.4 (36.1–59.8)
23F	1M post-pri	89.8 (84.5–93.7)	1.2 (1.0–1.5)	87.2 (81.4–91.7)	980.7 (689.0–1396.0)
	pre-bst	84.9 (79.0–89.8)	0.7 (0.6–0.8)	58.6 (50.8–66.1)	77.7 (50.6–119.3)
**Vaccine-related serotypes**					
6A	1M post-pri	29.6 (23.1–36.7)	0.1 (0.1–0.1)	26.1 (19.9–33.2)	12.9 (9.5–17.5)
	pre-bst	38.7 (31.7–46.1)	0.1 (0.1–0.2)	27.2 (20.7–34.4)	13.6 (9.9–18.7)
19A	1M post-pri	58.4 (50.9–65.6)	0.2 (0.2–0.3)	34.4 (27.6–41.8)	12.3 (9.5–15.7)
	pre-bst	49.5 (42.1–56.9)	0.2 (0.2–0.2)	15.7 (10.7–21.9)	6.4 (5.3–7.6)
**Protein D**					
		**% children with Ab conc ≥100 EL.U/mL (95% CI)**	**GMC (95% CI)**		
	1M post-pri	100 (98.0–100)	3822.7 (3457.1–4226.8)	NA	NA
	pre-bst	100 (98.0–100)	948.6 (841.2–1069.8)	NA	NA

The 3 primary PHiD-CV doses were administered at age 6, 10, and 14 weeks. 1M post-pri = 1 month post-primary (age 18 weeks); pre-bst = pre-booster (age 9–10 months).

Ab conc, antibody concentration; ATP, according-to-protocol; CI, confidence interval; ELISA, enzyme-linked immunosorbent assay; EL.U, ELISA units; GMC, geometric mean concentration; GMT, geometric mean titre; HUU, HIV-unexposed-uninfected children; IgG, Immunoglobulin G; NA, not applicable; OPA, opsonophagocytic activity.

Vaccination with 10-valent pneumococcal conjugate vaccine in infants according to HIV status

By Shabir A. MADHI *et al.*

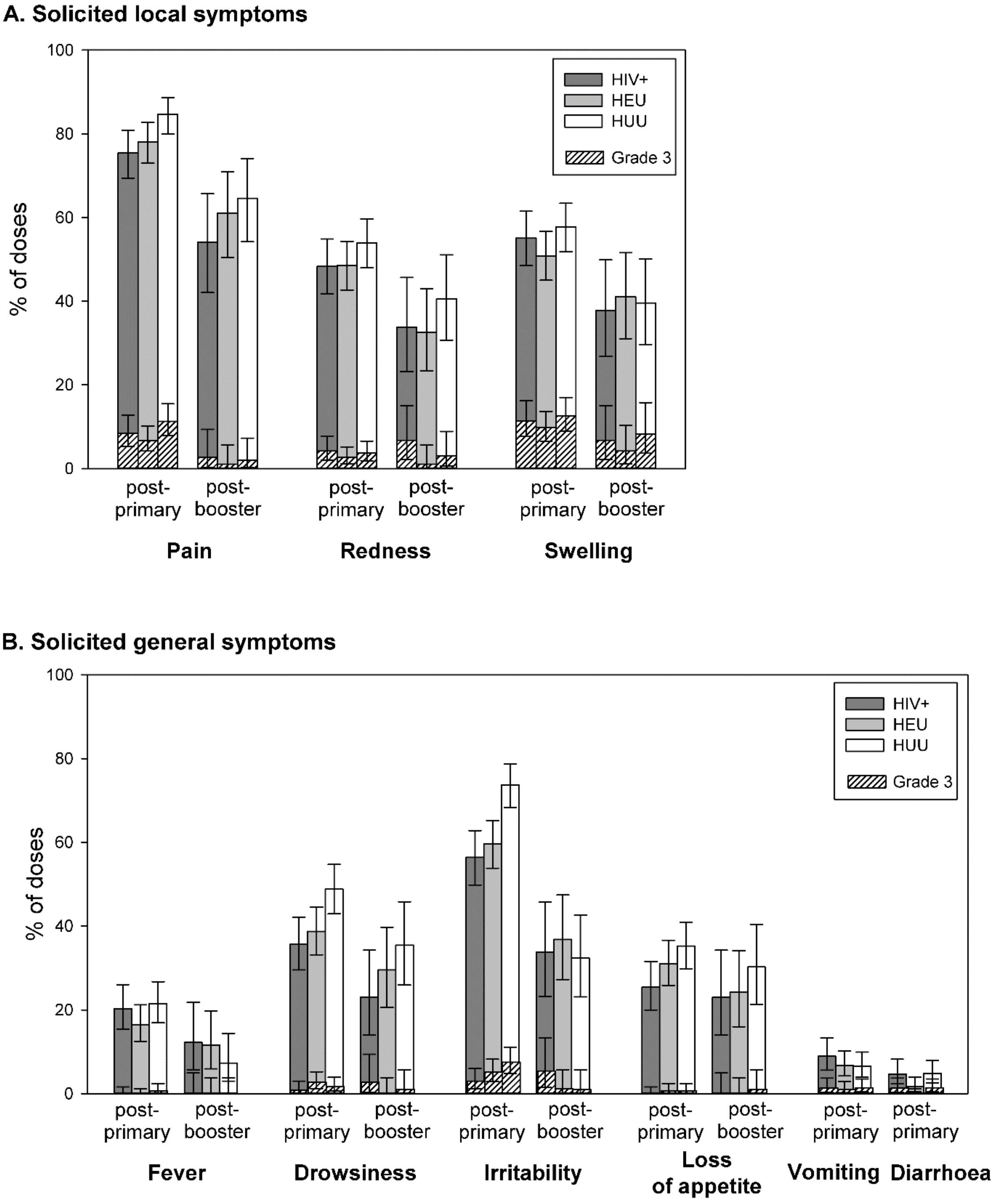

Supplemental Digital Content 5. Solicited local and general symptoms (overall per dose; total vaccinated cohort). HEU, HIV-exposed-uninfected; HIV+, HIV-infected; HUU, HIV-unexposed-uninfected. Error bars indicate 95% confidence interval. Grade 3 solicited symptoms were redness or swelling at the injection site with a diameter >30 mm; for pain, crying when the limb was moved or the limb being spontaneously painful; for fever, axillary temperature ≥39.5°C; for drowsiness, preventing normal activity; for irritability, crying that could not be comforted or prevented normal activity; for loss of appetite, not eating at all; for vomiting, ≥3 episodes of vomiting (≥1 hour after feeding) per day and for diarrhoea, ≥6 looser than normal stools per day; (vomiting and diarrhoea were only assessed post-priming).
